# Supplementary material for: Neural effects of continuous theta-burst stimulation in macaque parietal neurons
Source: eLife. 2022 Sep 13;11:e65536. doi: 10.7554/eLife.65536 (PMC9470151; doi:10.7554/eLife.65536)
Supplement: Transparent reporting form [file elife-65536-transrepform1.docx]

***eLife’s* transparent reporting form**

We encourage authors to provide detailed information *within their submission* to facilitate the interpretation and replication of experiments. Authors can upload supporting documentation to indicate the use of appropriate reporting guidelines for health-related research (see [EQUATOR Network](http://www.equator-network.org/%20)), life science research (see the [BioSharing Information Resource](https://biosharing.org/)), or the [ARRIVE guidelines](http://www.plosbiology.org/article/info:doi/10.1371/journal.pbio.1000412) for reporting work involving animal research. Where applicable, authors should refer to any relevant reporting standards documents in this form.

If you have any questions, please consult our Journal Policies and/or contact us: [editorial@elifesciences.org](mailto:editorial@elifesciences.org).

**Sample-size estimation**

- You should state whether an appropriate sample size was computed when the study was being designed
- You should state the statistical method of sample size computation and any required assumptions
- If no explicit power analysis was used, you should describe how you decided what sample (replicate) size (number) to use

Please outline where this information can be found within the submission (e.g., sections or figure legends), or explain why this information doesn’t apply to your submission:

As in almost all single-cell studies in monkeys, we did not calculate the sample size. In stead, we acquired a substantial number of neurons in one animal (N=51 neurons), and replicated the results in a second animal (N=35). Moreover, because we recorded neurons for a variable time interval after stimulation onset (as long as the spike waveform was still present), we could not predict how many neurons would contribute to each time interval.

**Replicates**

- You should report how often each experiment was performed
- You should include a definition of biological versus technical replication
- The data obtained should be provided and sufficient information should be provided to indicate the number of independent biological and/or technical replicates
- If you encountered any outliers, you should describe how these were handled
- Criteria for exclusion/inclusion of data should be clearly stated
- High-throughput sequence data should be uploaded before submission, with a private link for reviewers provided (these are available from both GEO and ArrayExpress)

Please outline where this information can be found within the submission (e.g., sections or figure legends), or explain why this information doesn’t apply to your submission:

We recorded 51 neurons in one monkey, and 35 neurons in a second animal (see page 10 first paragraph of results section).

**Statistical reporting**

- Statistical analysis methods should be described and justified
- Raw data should be presented in figures whenever informative to do so (typically when N per group is less than 10)
- For each experiment, you should identify the statistical tests used, exact values of N, definitions of center, methods of multiple test correction, and dispersion and precision measures (e.g., mean, median, SD, SEM, confidence intervals; and, for the major substantive results, a measure of effect size (e.g., Pearson's r, Cohen's d)
- Report exact p-values wherever possible alongside the summary statistics and 95% confidence intervals. These should be reported for all key questions and not only when the p-value is less than 0.05.

Please outline where this information can be found within the submission (e.g., sections or figure legends), or explain why this information doesn’t apply to your submission:

We always used non-parametric statistics (Wilcoxon ranksum test) on the neuronal responses so that no assumptions about the distributions had to be made. We also provide the percent reduction in neuronal response for each time interval at the population level (Figure 4 and text on pages 12-13).

On the behavioral data, we calculated one-way ANOVAs (the effect of no-, low- and high stimulation), and repeated measures ANOVAs with correction for multiple comparisons with factors time and stimulation. This is described in Methods on page 8-9.

Because the number of trials in each time epoch varies for every neuron recorded, it was not very practical to report the exact number of observations for each test. We report the exact p-values throughout the manuscript, and F and eta square values for the ANOVAs. Table 1 (page 12) gives the exact numbers of neurons (for the two animals separately and for the combined data) showing significant effects for every time interval post cTBS.

(For large datasets, or papers with a very large number of statistical tests, you may upload a single table file with tests, Ns, etc., with reference to sections in the manuscript.)

**Group allocation**

- Indicate how samples were allocated into experimental groups (in the case of clinical studies, please specify allocation to treatment method); if randomization was used, please also state if restricted randomization was applied
- Indicate if masking was used during group allocation, data collection and/or data analysis

Please outline where this information can be found within the submission (e.g., sections or figure legends), or explain why this information doesn’t apply to your submission:

Not applicable

**Additional data files (“source data”)**

- We encourage you to upload relevant additional data files, such as numerical data that are represented as a graph in a figure, or as a summary table
- Where provided, these should be in the most useful format, and they can be uploaded as “Source data” files linked to a main figure or table
- Include model definition files including the full list of parameters used
- Include code used for data analysis (e.g., R, MatLab)
- Avoid stating that data files are “available upon request”

Please indicate the figures or tables for which source data files have been provided:

We provide an excel file with the p values for each neuron recorded and Matlab code for data analysis.
